# Biological activity of new bioactive steroids deriving from biotransformation of cortisone

**DOI:** 10.1186/s12934-022-01967-2

**Published:** 2022-11-24

**Authors:** Stefania Costa, Paola Tedeschi, Luca Ferraro, Sarah Beggiato, Alessandro Grandini, Stefano Manfredini, Raissa Buzzi, Gianni Sacchetti, Giuseppe Valacchi

**Affiliations:** 1 Department of Chemical, Pharmaceutical and Agricultural Sciences, University of Ferrara, Via L. Borsari, 46 Ferrara, 44121 Ferrara, Italy; 2 Department of Life Sciences and Biotechnology, University of Ferrara, Via L. Borsari, 46 Ferrara, 44121 Ferrara, Italy; 3 Laboratory for Technologies of Advanced Therapies (LTTA), University of Ferrara, Via Fossato Di Mortara 70, 44121 Ferrara, Italy; 4 Department of Environmental Sciences and Prevention, University of Ferrara, Via L. Borsari, 46 Ferrara, 44121 Ferrara, Italy; 5 North Carolina Research Campus, Plants for Human Health Institute, Animal Science, North Carolina State University, Kannapolis, NC 28081 USA; 6 Department of Food and Nutrition, Kyung Hee University, Seoul, 02447 Korea

**Keywords:** Biotransformations, Cortisone, Steroids, Neuroprotective, Anti-inflammatory, Antioxidant

## Abstract

Cortisone is a metabolite belonging to the corticosteroid class that is used pharmaceutically directly as a drug or prodrug. In addition to its large consumption, its use is linked to several side effects, so pharmaceutical research aims to develop effective drugs with low or no side effects, alternative compounds to cortisone are part of an active investment in ongoing research on drug discovery. Since biotransformation can be considered a source of new molecules with potential therapeutic use, the present work focuses on a preliminary in vitro study aimed at evaluating the mutagenic, anti-inflammatory, antioxidant and neuroprotective activity of SCA and SCB molecules obtained from the biotransformation of cortisone using *Rh. Rhodnii* strain DSM 43960. The results obtained are very encouraging due to the safety of biotransformed compounds with reference to genotoxicity checked by Ames test, to the very high antioxidant capacity and to the anti-inflammatory activity. In fact, thecompounds inhibited both the TNFα-stimulated expression and secretion of NFkB target cytokines, and COX activity, and can activate the glucocorticoid receptor. Finally SCA and SCB exhibited neuroprotective properties.

## Introduction

Corticosteroids are natural steroid hormones produced by the adrenal cortex, classified as glucocorticoids and mineralocorticoids. Glucocorticoids control a variety of physiologic processes, thus exerting a primary role in regulating carbohydrate metabolism, and displaying immunosuppressant, anti-inflammatory, and vasoconstrictive properties. Mineralocorticoids, instead, modulate electrolyte concentrations and, consequently, the body water content by influencing ion transport in the epithelial cells of the renal tubules [1]. Since their discovery 70 years ago, corticosteroids have been shown to be useful therapeutic compounds [2]. In fact, corticosteroid-based therapy is used for the treatment of several pathological conditions, including infectious and inflammatory disorders, allergic and autoimmune diseases, increased water excretion, pathological hypoglycemia, as well as neurological, hematological and skin disorders [1, 3]. Glucocorticoids mainly act by interacting with specific receptors (i.e. glucocorticoid receptors), thus regulating either genomic and non-genomic pathways. The genomic action of the activated glucocorticoid receptor controls the expression of many genes, such as proinflammatory mediators and transcription factors (e.g. cytokines, chemokines and adhesion molecules). These effects contribute to the anti-inflammatory and immunoregulatory actions of glucocorticoids [4, 5].

Despite their undoubted efficacy in treating various pathologies, corticosteroids have various side effects that limit their use. As a consequence, in the last few decades, there has been an intense research activity focused on the development of new steroid drugs without corticosteroid serious adverse effects. In this context, it is known that also minimal structural differences of these molecules can significantly change their potency, efficiency, and half-life, as well as markedly improve their side effects. However, in 2021, the global corticosteroid market has an estimated revenue of 4.56 billion dollars, and it is expected to grow at a compound annual growth rate (CAGR) of 2%, also as a consequence of COVID-19 pandemic. As reported in the Corticosteroids Global Market Report 2021, it is therefore estimated that the market will reach 5.33 billion dollars in 2025 with a CAGR of 4%.

Microbiological transformations are an effective tool for the preparation of various molecules very difficult to synthesize with traditional chemical approaches [6]. Biotransformations by different isolated microorganisms or enzymes have been widely used in the bioconversion of steroid molecules, thus representing one of the best examples of the applications of this strategy as a sustainable approach to pharmaceutical industry processes [7]. Compared to traditional chemical synthesis, biotransformations, in line with the principles of green chemistry, have in fact the undoubtable advantage of a low environmental and economic impact on the production process. A further advantage of this technique lies in the selectivity of the enzymes catalyzing the reactions. In fact, the bioconversion can be carried out in a position of the steroid molecule that is difficult to access by chemical reagents and stereospecifically without the need for protection-deprotection steps of the functional groups. Thanks to this latter advantage and to the fact that it is possible to operate with multi-enzymatic complexes, a one-step transformation process can be developed.

Several examples of steroid molecule microorganisms-catalyzed biotransformations are reported in the scientific literature, some of which have been included in the synthesis processes of new pharmacological compounds [8–11]. The different functionalizations carried out on steroid molecules are oxidations, hydroxylations, redox and oxidation reactions of Baeyer–Villiger. These reactions, together with many others, can be performed as an alternative to chemical synthesis, by means of biocatalytic procedures [12]. It is also reported that some steroid biotransformation products display therapeutic advantages when compared to their starting substrates [13]. By biotransformations of cortisone and hydrocortisone catalyzed by *Rhodoccoccus coprophilus* and following a Δ1-dehydrogenation reaction, prednisone and prednisolone were respectively obtained, with interesting quantitative bioconversion yields. In these biotransformations, the formation of modified steroids has also been demonstrated at the level of the carbonyl present in position C_20_ of the side chain of the steroid nucleus [14].

**Scheme 1.**
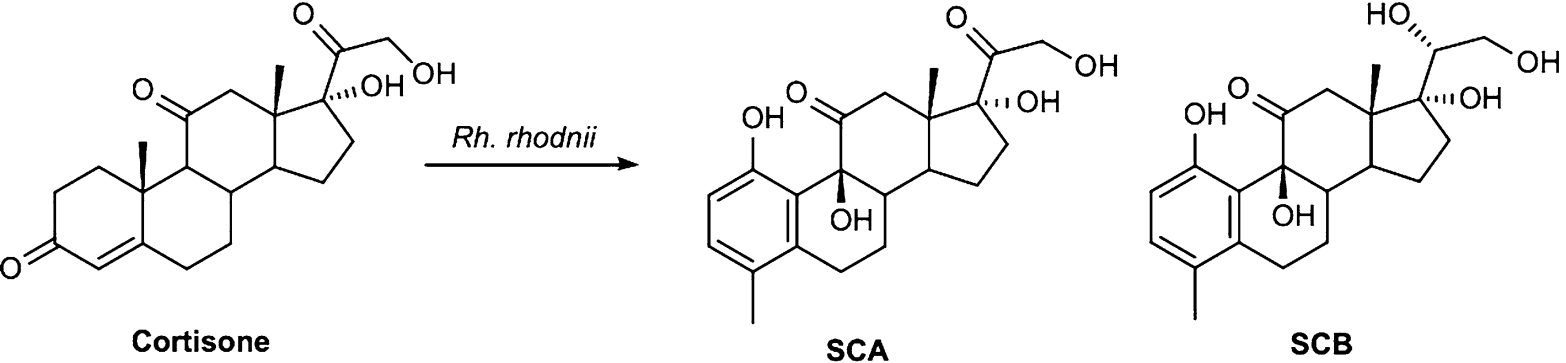
New steroids obtained by biotransformation of cortisone with *Rh. rhodnii*

In a previous work the biotransformation of cortisone with *Rhodococcus rhodnii* DSM 43960 afforded two new steroids, *i.e.*, 1,9β,17,21‐tetrahydoxy‐4‐methyl‐19‐nor‐9β‐pregna‐1,3,5(10)‐trien‐11,20‐dione (**SCA**) and 1,9β,17,20β,21‐ pentahydoxy‐4‐methyl‐19‐nor‐9β‐pregna‐1,3,5(10)‐trien‐11‐one (**SCB**) [15] as shown in Scheme 1.

In order to obtain, for the first time, indications of the possible therapeutic use of these compounds, in the present in vitro study the biological activities of SCA and SCB compounds have been evaluated for their putative anti-inflammatory, antioxidant and neuroprotective properties, as well as their genotoxic safety.

## Results

### Mutagenicity assay (Ames test)

SCA and SCB compounds were assayed for genotoxicity evaluation through the Ames test employing the histidine-requiring *Salmonella typhimurium* mutant TA98 and TA100 strains allowing to check frameshift mutation and base-pair substitution, with and without metabolic activation induced by S9 Mix [16–19].

The results obtained are reported in Tables 1, 2. For none of the samples genotoxic activity emerged at all the SCA or SCB concentrations tested with all the *Salmonella* strains.

**Table 1:** Ames test performed for SCA employing the histidine-requiring *Salmonella typhimurium* mutant TA98 and TA100 strains

	*Salmonella typhimurium* TA98						*Salmonella typhimurium* TA100					
	− S9			+ S9			− S9			+ S9		
	average	SD	t/c	average	SD	t/c	average	SD	t/c	average	SD	t/c
DMSO	20,7	5,1	**1.0**	37,3	3,2	**1.0**	165.7	6.8	**1.0**	181.0	7.0	**1.0**
C +	1141,7	99,1	**55.2**	1302,0	59,2	**34.9**	1001.7	56.1	**6.0**	2477.3	110.0	**13.7**
1	17,7	5,5	**0.9**	43,7	5,9	**1.2**	163.0	8.5	**1.0**	179.3	15.2	**1.0**
5	23,7	2,1	**1.1**	43,3	3,8	**1.2**	164.3	6.1	**1.0**	181.7	9.6	**1.0**
10	23,3	2,5	**1.1**	41,3	3,5	**1.1**	158.0	5.0	**1.0**	185.3	14.7	**1.0**
50	25,3	4,5	**1.2**	39,3	2,9	**1.1**	157.0	7.0	**0.9**	205.7	10.5	**1.1**
100	20,3	2,1	**1.0**	41,3	2,9	**1.1**	166.7	5.0	**1.0**	194.3	8.0	**1.1**

**t/c** number of revertant colonies in treated plates/number of revertant colonies in untreated plates

^a^Significant values according to Ames computation

*DMSO, C +* : negative and positive controls respectively, *SD* standard deviation

**Table 2:** Ames test performed for SCB employing the histidine-requiring *Salmonella typhimurium* mutant TA98 and TA100 strains

	*Salmonella typhimurium* TA98						*Salmonella typhimurium* TA100					
	− S9			+ S9			− S9			+ S9		
	average	SD	t/c	average	SD	t/c	average	SD	t/c	average	SD	t/c
DMSO	24	4	**1.0**	32	4	**1.0**	166	10	**1.0**	191	15	**1.0**
C +	1142	99	**47.0**	1302	59	**40.0**	1002	56	**6.0**	2477	110	**13.0**
1	24	5	**1.0**	34	4	**1.1**	157	9	**0.9**	190	9	**1.0**
5	23	5	**0.9**	35	3	**1.1**	169	11	**1.0**	194	10	**1.0**
10	22	4	**0.9**	35	5	**1.1**	164	7	**1.0**	193	12	**1.0**
50	21	5	**0.9**	31	5	**1.0**	169	11	**1.0**	189	9	**1.0**
100	19	2	**0.8**	30	5	**0.9**	180	11	**1.1**	195	8	**1.0**

**t/c**: number of revertant colonies in treated plates/number of revertant colonies in untreated plates

^a^Significant values according to Ames computation

*DMSO, C + * negative and positive controls respectively, *SD*: Standard deviation

In the following tables are shown results of Ames test performed for SCA and SCB employing the histidine-requiring *Salmonella typhimurium* mutant TA98 and TA100 strains.

### Determination of antioxidant properties

**Fig. 1:**
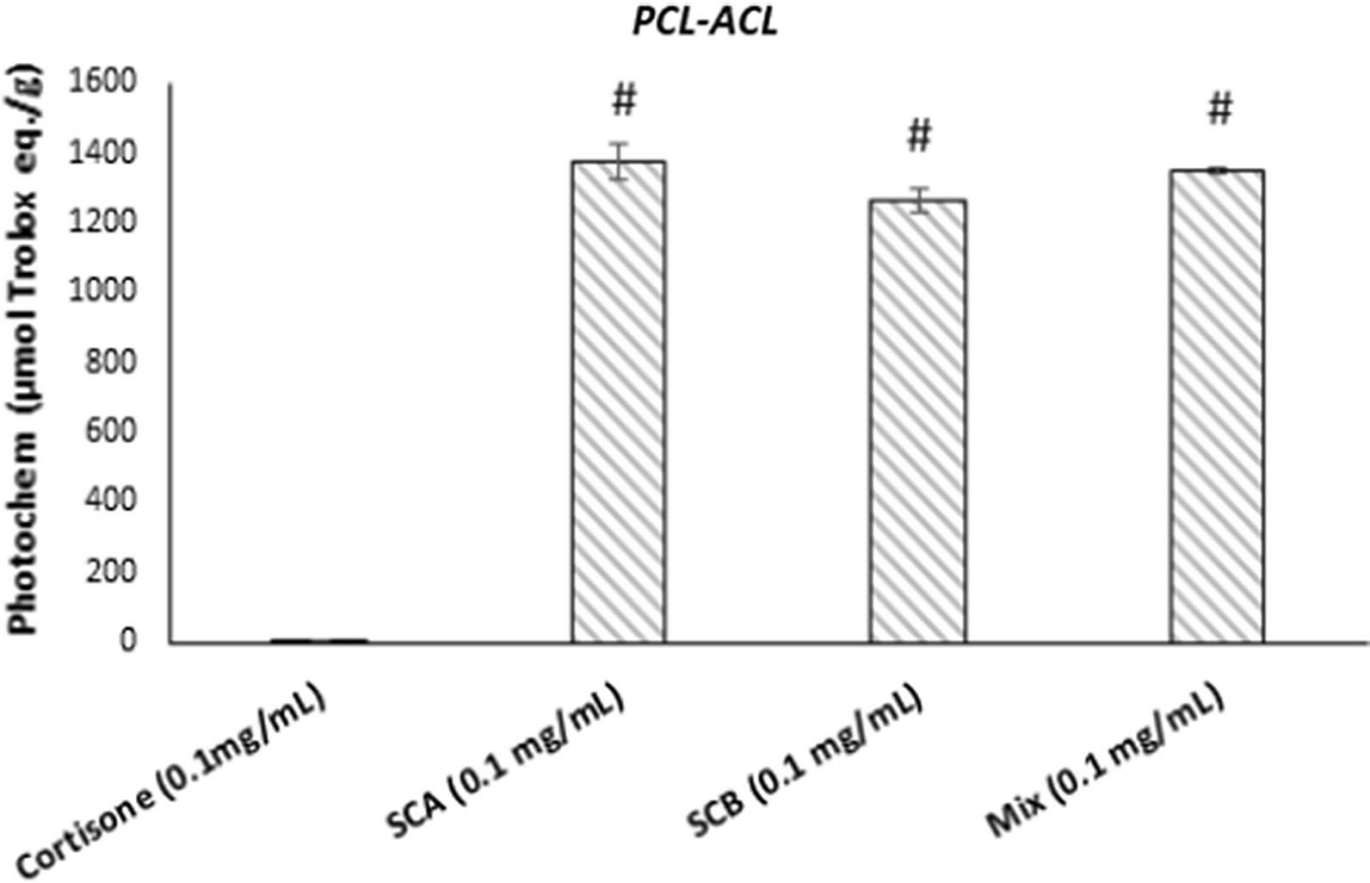
Antioxidant activity by PCL-ACL assay of cortisone, SCA, SCB and mix (0.1 mg/mL). Data are expressed as µg of Trolox equivalents for g of samples. Values represent mean ± SEM. Data are representative of three independent experiments. Statistical significance determined by one-way ANOVA. #p < 0.05, significantly different from control values (cortisone)

**Fig. 2:**
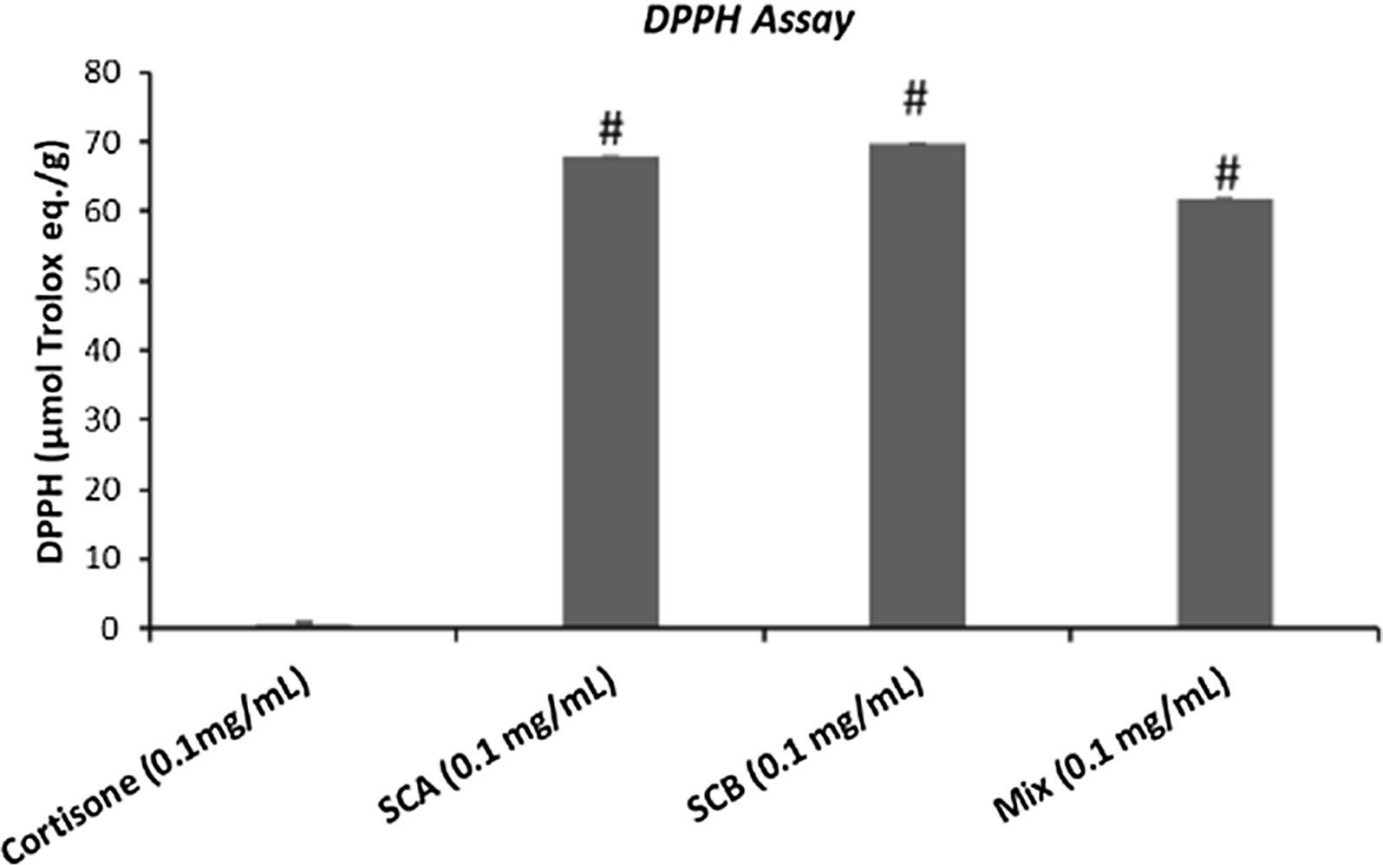
DPPH radical scavenging activity of cortisone (control), SCA, SCB and mix (0.1 mg/mL). Data are expressed as µg of Trolox equivalents for g of samples (mean ± SEM). Data are representative of three independent experiments. Statistical significance determined by one-way ANOVA. #p < 0.05, significantly different from control values (cortisone)

The antioxidant properties of control (cortisone) and SCA and SCB compounds (0.1 mg/mL) and their relative mix were evaluated with two methods, i.e. by means PCL-ACL assay (Fig. 1) and DPPH·radical scavenging activity (Fig. 2). In both cases, the results are expressed as µg of Trolox equivalents per g of samples. As shown in Figs. 1 and 2, the antioxidant activity plots of SCA, SCB and mix were higher for SCA and SCB against the control. By focusing on results obtained with PCL-ACL assay, SCA and SCB presented a very high antioxidant capacity (respectively 1375 and 1265 µmol/g), while the control (cortisone) did not display antioxidant capacity (0.13 µmol/g). It was interesting to point out how the mix presented an intermediate antioxidant activity.

Even if the data obtained with DPPH· test were lower than those provided by PCL-ACL assay, the two molecules obtained from the biotransformation of cortisone with *Rhodococcus rhodnii,* however presented a high antioxidant property compared with the cortisone precursor. The mix obtained from the two products with this test showed a slightly lower value than SCA and SCB individually tested.

### SCB partially prevented TNFα-induced p65 NFκB nuclear translocation

To evaluate the anti-inflammatory activity of SCA and SCB compounds, we first examined their potential effects in preventing TNFα-induced activation of the transcription factor NFκB. For this purpose, HaCaT cells were pretreated for 24 h with 10 μM of SCA and SCB and then challenged with 10 ng/mL TNFα for 1 h to activate NFκB signaling pathway.

**Fig. 3:**
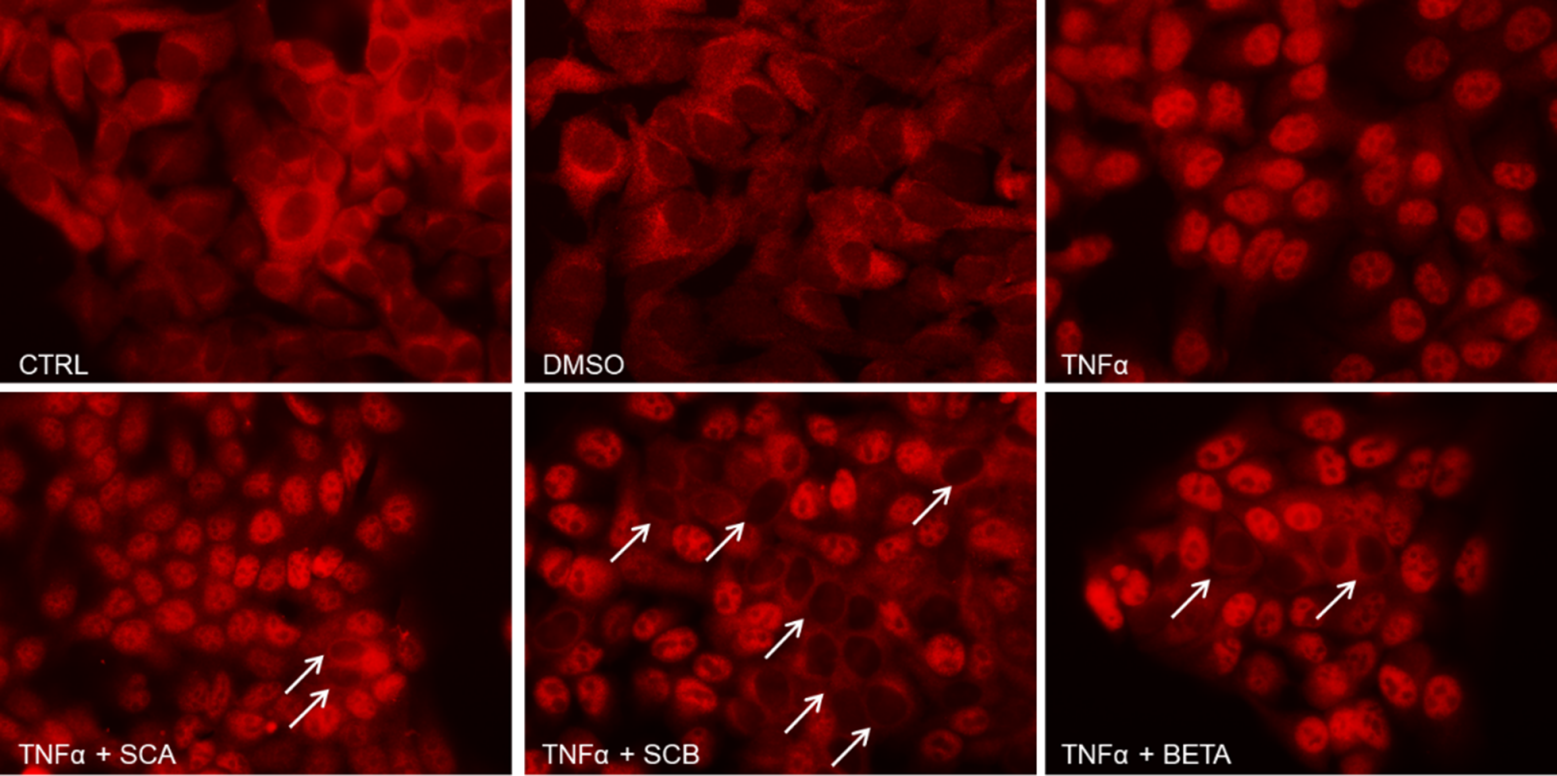
Potential anti-inflammatory effect of SCA and SCB in preventing TNFα-mediated NF-κB p65 nuclear translocation in HaCaT cells**.** Twenty-four hours after the treatment with 10 μM of SCA and SCB, HaCaT cells were stimulated with 10 ng/mL TNFα for 1 h. Then, p65 NF-κB nuclear translocation was assessed by immunofluorescence. DMSO- and betamethasone-treated cells were included in the assay as negative (vehicle) and positive controls, respectively. White arrows indicate some p65-negative nuclei. Representative images are shown. Original magnification: ×400

As shown in Fig. 3, TNFα stimulation induced a strong nuclear accumulation of p65 NF-κB subunit in HaCaT cells. While SCA barely affected TNFα-stimulated translocation of p65 to the nucleus, SCB pretreatment showed the ability in counteracting the proinflammatory stimulus, as indicated by the increased numbers of HaCaT cells with p65-negative nuclear staining (Fig. 3). Similarly, pretreatment of HaCaT cells with betamethasone, a well-known steroidal anti-inflammatory agent, only partly prevented TNFα-mediated p65 activation (Fig. 3). As expected, vehicle-treated (DMSO) and untreated HaCaT cells displayed the immunofluorescence staining of p65 mostly restricted in the cytoplasm (Fig. 3).

### SCA and SCB inhibited the TNFα-stimulated expression and secretion of NFκB target cytokines

We further investigated the potential anti-inflammatory effects of SCA and SCB compounds by evaluating gene and protein expression of NFκB target cytokines in HaCaT cells stimulated with TNFα.

**Fig. 4:**
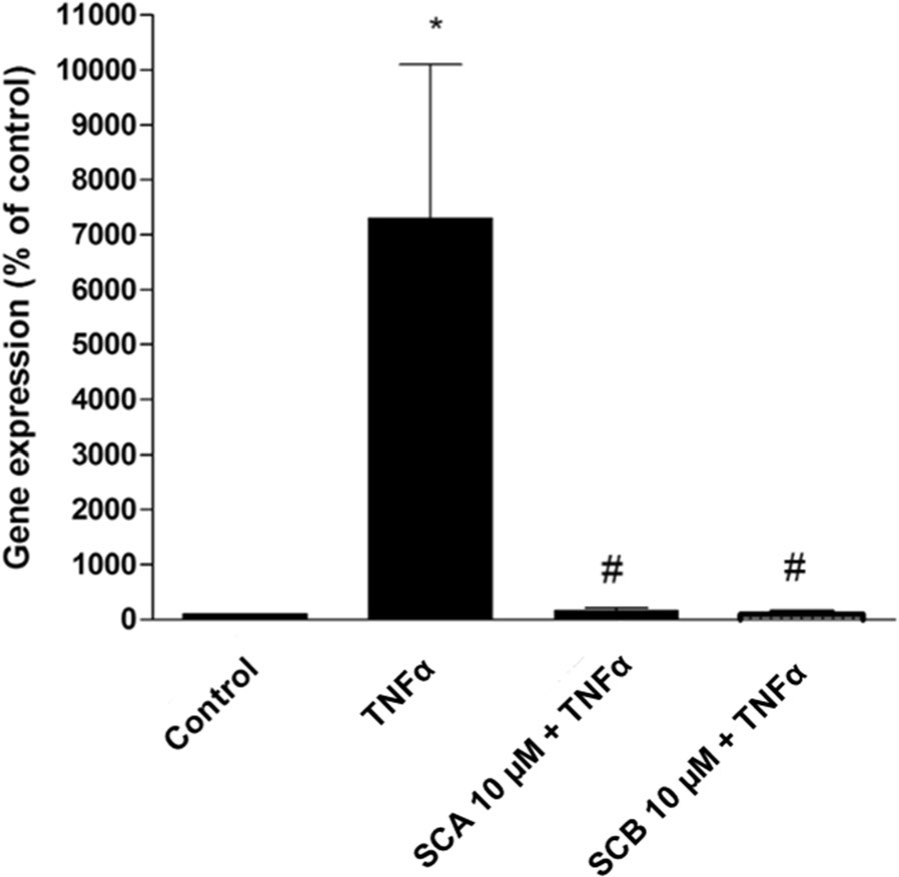
Fig. 4. Effect of SCA and SCB on IL-8 gene expression in TNFα-stimulated HaCaT cells. TNFα-induced IL-8 gene expression was evaluated by real-time PCR in total RNA extracts from HaCaT cells either treated or un-treated for 24 h with 10 μM of SCA and SCB. Values represent mean ± SEM. Data are representative of three independent experiments. Statistical significance determined by two-way ANOVA. *vs control (DMSO); ^#^vs TNFα treated samples; p < 0.05

In addition to the induction of p65 nuclear translocation (Fig. 3), the treatment of keratinocytes with 10 ng/mL TNFα for 1 h also promoted a significant increase in the gene expression of the proinflammatory cytokine IL-8 (Fig. 4). Of note, pretreatment for 24 h with both SCA and SCB compounds (10 µM) was able to completely abolish TNFα-triggered IL-8 mRNA upregulation (Fig. 4).

**Fig. 5:**
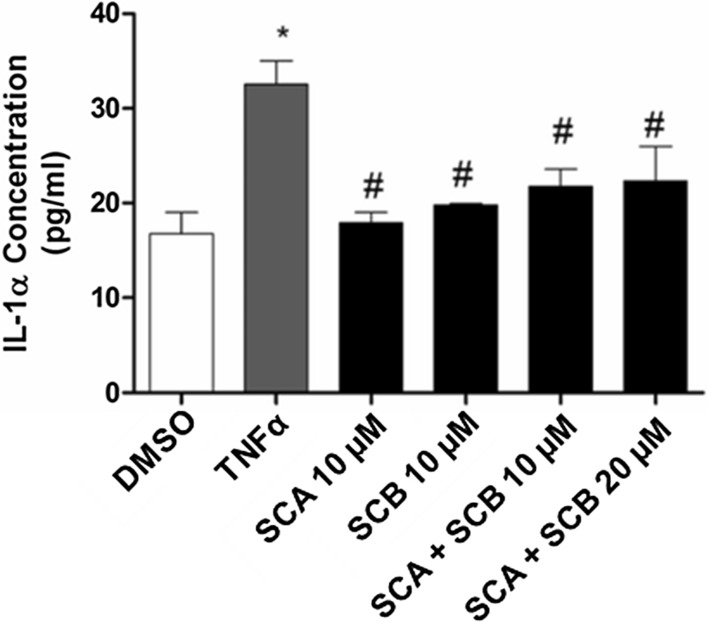
Fig. 5. Effect of SCA and SCB on IL-1α secretion in TNFα-stimulated HaCaT cells. HaCaT cells were either treated or un-treated with 10 μM of SCA and SCB or the combined mix of the two compounds at either 10 and 20 μM doses. After 24 h, cells were stimulated with 10 ng/mL TNFα for 1 h. ELISA assay was used to determine IL-1α release in the cell culture supernatants 24 h after the treatments. Values represent mean ± SEM. Data are representative of three independent experiments. Statistical significance determined by two-way ANOVA. *vs control (DMSO); ^#^vs TNFα treated samples; p < 0.05

Moreover, we also measured the release of IL-1α in cell supernatants by ELISA. In line with the real-time PCR data, stimulation of HaCaT cells with TNFα induced more than two-fold increase in IL-1α secretion compared to the control cells (i.e. vehicle treated cells) (Fig. 5). Consistently, TNFα-triggered IL-1α release was markedly inhibited by pretreatment with both SCA and SCB (Fig. 5).

Furthermore, we examined whether the two compounds might lead to additive or synergistic anti-inflammatory effects. However, as shown in Fig. 5, the combined treatment with SCA and SCB at two different concentrations (10 and 20 μM) did not induce any further decrease in IL-1α secretion from HaCaT cells.

### SCB partially inhibited COX activity

**Fig. 6:**
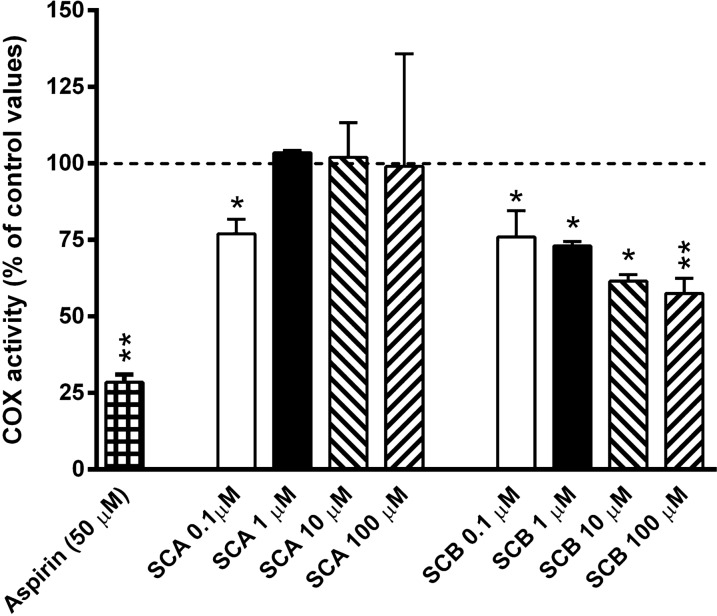
Effects of aspirin (acetylsalicylic acid; 50 µM), SCA (0.1–100 μM) and SCB (0.1–100 μM) on cyclooxygenase (COX) activity. Results are reported as % changes of control COX activity (dotted line). Values represent mean ± SEM. Data are representative of three independent experiments. Statistical significance was determined by one-way ANOVA followed by Bonferroni’s multiple comparison test. *p < 0.05, **p < 0.01 significantly different from control values (*i.e.* basal kit cox-activity)

To evaluate the possible mechanism(s) underlying the anti-inflammatory properties of SCA and SCB compounds, we firstly examined their potential effects in inhibiting COX enzymatic activity, a common target for anti-inflammatory drugs. As shown in Fig. 6, while SCA barely affected COX activity only at the lower concentration tested (0.1 µM), SCB treatment inhibited COX activity at all concentrations tested in a dose-dependent trend (0.1–10 µM). As expected, aspirin (50 µM) strongly inhibited COX activity.

### SCA and SCB activate the glucocorticoid receptor

**Fig. 7:**
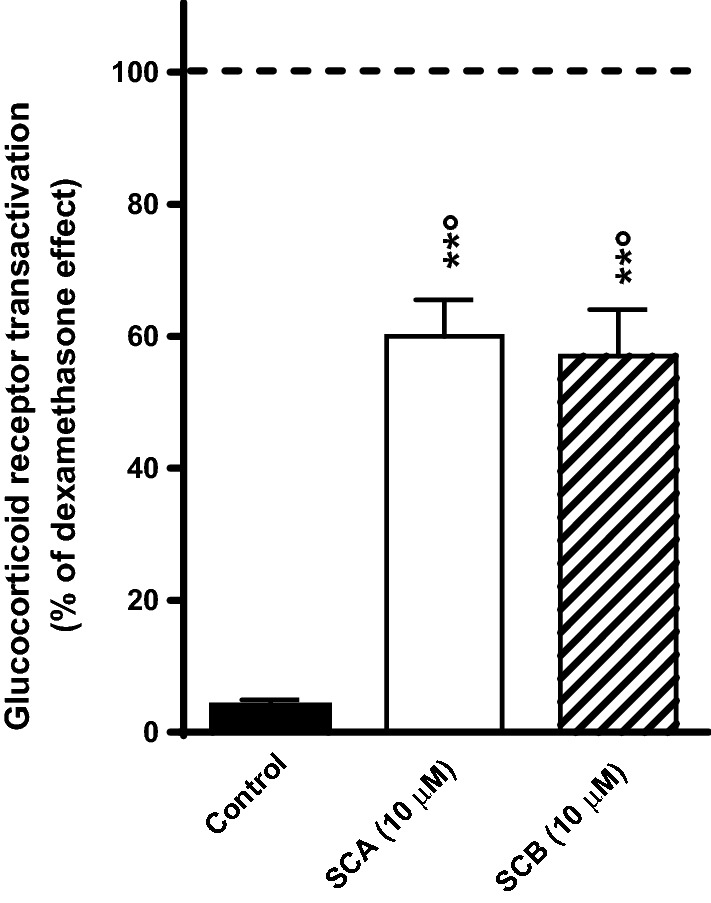
Effects of SCA (10 μM) and SCB (10 μM) on glucocorticoid receptor transactivation. Control group was treated with the vehicle (1% DMSO solution). Results are reported as % changes of the glucocorticoid receptor transactivation induced by dexamethasone (0.1 μM; dotted line). Values represent mean ± SEM. Data are representative of three independent experiments. Statistical significance was determined by one-way ANOVA followed by Bonferroni’s multiple comparison test. **p < 0.01 significantly different from control values; °p < 0.05 significantly different from dexamethasone group

To further evaluate the possible mechanism (s) underlying the anti-inflammatory properties of SCA and SCB compounds, we then evaluated their potential effects in activating the glucocorticoid receptor. As shown in Fig. 7, both SCA (10 µM) and SCB (10 µM) activated glucocorticoid receptors, demonstrating a similar potency. The effects of the compounds were, however, lower than that of dexamethasone (0.1 µM), used as a reference glucocorticoid receptor agonist.

### SCA and SCB display neuroprotective properties

**Fig. 8:**
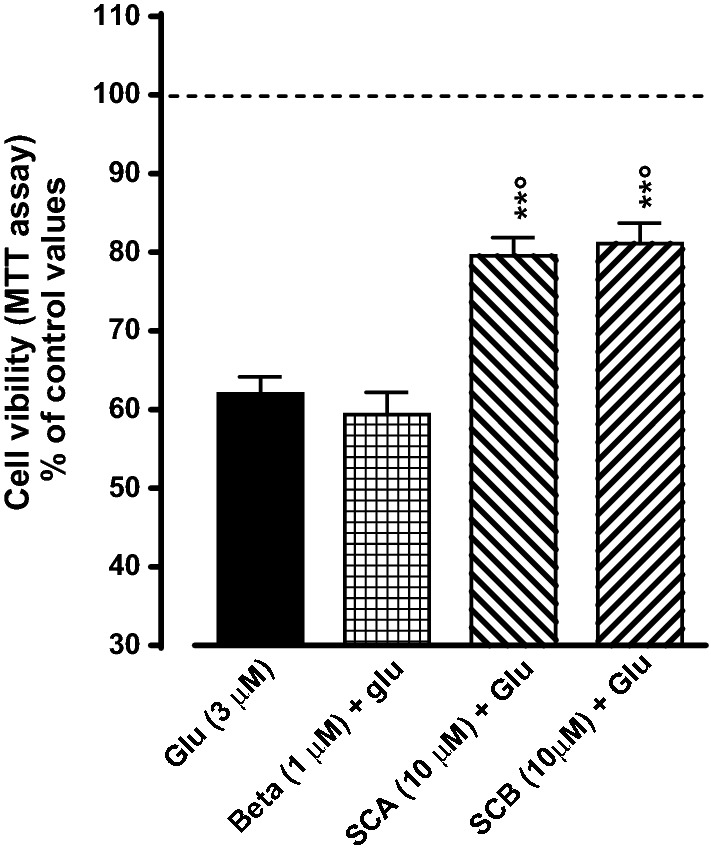
Effects of betamethasone (Beta; 1 μM), SCA (10 μM) and SCB (10 μM) on glutamate (Glu)-induced neurotoxicity in primary cultures of rat cerebral cortex neurons. Control group was treated with the vehicle (1% DMSO solution). Results are reported as % changes of control values (dotted line). Values represent mean ± SEM. Data are representative of five independent experiments. Statistical significance was determined by one-way ANOVA followed by Bonferroni’s multiple comparison test. **p < 0.01 significantly different from control values; p < 0.05 significantly different from dexamethasone group

The possible neuroprotective properties of SCA and SCB have also been evaluated, by measuring the effects of the compounds against glutamate-induced neurotoxicity in primary cultures of rat cerebral cortex neurons. As shown in Fig. 8a 24 h exposure to glutamate induced a reduction in cell viability in primary cultures of rat cerebral cortex neurons. The pre-treatment with SCA (10 μM) and SCB (10 μM) partially reduced the glutamate-induced neurotoxicity. On the contrary, the glucocorticoid receptor agonist betamethasone (1 μM) did not affect glutamate-induced neurotoxicity in primary cultures of rat cerebral cortex neuron (Fig. 8).

## Discussion

Industrial biotechnology comprises the application of biotechnology-based strategies to traditional industrial processes. Bioprocessing of starting materials by means of microbes, microorganisms, enzymes represents the basis of innovative processes that companies, researchers and scientists are seeking to develop for the production of commercial products, including the development of new chemical entities. In this context, the application of biotechnology at the industrial level has allowed it to replace many traditional processes based on chemical synthesis. The biotransformation approach follows the guidelines of *green-chemistry* as it works in mild reaction conditions and aqueous solvents. Furthermore, this microorganism-based approach allows to modify starting molecules exploiting several advantages over chemical synthesis, such as the regio- and/or stereospecificity of the reactions. Despite the exciting advantages, only a few biotransformation-based approaches have been applied in the pharmaceutical industry so far [20]. A recent study reported the production of two new steroid compounds (coded SCA and SCB), not chemically synthesizable, by the biotransformation of cortisone [15]. As corticosteroid-based therapies generate a wide range of side effects and the pharmaceutical industry is increasingly looking for compounds with therapeutic activity comparable to that of glucocorticoids but with fewer side effects, it became relevant to evaluate the possible pharmacological activities and therapeutic potential of these two new compounds. Thus, in the present in vitro study, the putative anti-inflammatory, antioxidant and neuroprotective properties, as well as possible genotoxicity of SCA and SCB compounds, have been evaluated.

Genotoxicity testing of new pharmaceuticals prior to their commercialization is required by regulatory agencies worldwide, and the evaluation of possible genetic toxicity is obviously fundamental to make informed decisions with respect to risk associated with the human exposure to new compounds. Based on these considerations and in view of the possibility to propose the development of SCA and SCB as new pharmaceuticals, the two compounds were firstly assayed for genotoxicity evaluation through the Ames test, which is generally considered an appropriate preliminary screening tool to determine the mutagenic potential of new chemicals for different industrial applications, from agro-chemicals, pharmaceuticals and medical devices to health products (for *e.g.* personal cares, cosmetics, nutraceuticals, etc.). For the high significant predictive capacity, Ames test is required by the regulatory agencies for registration or acceptance of new compounds [21], and is based on the use of numerous *Salmonella* strains, specially engineered to detect mutagenic activity, each one with a different mechanism and sensitivity. The histidine-requiring *Salmonella typhimurium* mutant strains used to evaluate SCA and SCB mutagenicity are particularly appropriate to preliminary check genotoxic potential of new chemical compounds because both of them have a plasmid that induce an increasing of error-prone repair of DNA damage (pKM101), and mutations that induce an increased permeability to larger molecules through a defective lipopolysaccharide layer (rfa mutations) and avoid the possibility to repair excision DNA damage (uvrB mutations) [21, 22]. These genetic properties allow to check frameshift mutation and base-pair substitution through the increasing number of revertant colonies in presence of mutagenic chemical compounds, with and without metabolic activation induced by S9 mix. The results obtained clearly excluded possible mutagenicity of SCA and SCB compounds as evaluated by means of Ames test. This is undoubtedly an encouraging result, although generally a three or four-test battery, also including in vitro mammalian mutagenesis, in vitro chromosome aberration analysis and an in vivo chromosome stability assay, is required to fully evaluate the genotoxic potential of a chemical compound.

Mooradian in a study of 14 steroids showed that cortisone and corticosterone appeared to have very mild pro-oxidant properties [23]. Other steroids tested such as estrone, testosterone, progesterone, androstenedione, dehydroepiandrosterone, cortisol, tetrahydrocortisone, deoxycorticosterone and aldosterone had no significant antioxidant properties, while estrogens especially estriol and 17 β-estradiol are naturally occurring antioxidants [24–26]. Thus, the antioxidant properties of SCA and SCB have been assessed and compared to that of cortisone. By focusing on results obtained with both antioxidant tests, PCL-ACL assay and DPPH, it is evident that SCA and SCB display very good antioxidant properties, unlike cortisone. It is also interesting that the mixture of the two compounds has, as expected, a high antioxidant capacity with respect to the single compounds. Comparing the data obtained by the two methods, higher total antioxidant properties of SCA and SCB were observed with Photochem test. This significative differences between the two methods can be due to the fact that the DPPH radical had a higher molecular weight and so steric hindrance when compared with superoxide anion radical. This difference may preclude the interaction with some substrate molecules such as the analyzed compounds. Overall, however, both methods confirmed the great antioxidant capacity of SCA and SCB; in particular, the data provided by PCL method showed the ability of SCA and SCB to counteract the dangerous superoxide radical with an antioxidant capacity around 1000% the cortisone one.

Considering the steroid structure of SCA and SCB, as well as the well-characterized cross talk between oxidative stress and inflammation, as recently defined OxInflammation [27], the putative anti-inflammatory properties of the two compounds have been also tested. The results demonstrate the ability of both compounds to prevent a proinflammatory response measured by IL-1 and IL-8 levels and NFkB activation.

Activation of the canonical NF-κB pathway relies on its ability to translocate to the nucleus and bind with DNA controlled by the inhibitor of κB (IκB). IκB protein can bind NF-κB dimers to prevent the activation of NF-κB. The most common member of IκB in mediating the activation of the typical NF-κB pathway is IκBα, which plays a central role in subsequent transcription. Degradation of IκBα involves phosphorylation of IκB kinase. In response to external stimulation, NF-κB-bound IκB protein undergoes specific phosphorylation, ubiquitination, and proteasome-mediated proteolysis, allowing NF-κB to localize to the nucleus and bind to DNA and transcribe for inflammatory genes including IL-1a and IL-8. At this stage we do not know the exact mechanism involved in NF-κB inhibition by SCA or SCB. It could be that SCA and SCB are able to prevent IkB phosphorylation as suggested for cortisol, [28] or even activate the antioxidant master regulator Nrf2 which has been shown to compete with NF-κB for similar DNA binding sequences [29]. Being NF-κB a redox sensitive master regulator transcription factor involved in the inflammatory responses, its inhibition by SCA and SCB could be a consequence of their antioxidant properties and this could explain the effect on IL-1 gene expression. Indeed it has been well demonstrated that ROS and H_2_O_2_ can be key molecules for NF-κB activation [30] and lead to IL-1 and IL-8 secretion. The antioxidant properties (either direct or indirect) of SCA and SCB could prevent endogenous ROS production or quench exogenous ROS levels so to prevent NF-κB activation and the subsequent cytokines transcription. Although the loop between oxidative damage and activation of inflammatory response is still a “chicken and egg puzzle” the inhibition of one of the two mechanisms can quench the activation of the other one, leading to a lower inflammation and oxidative damage. In view of these data and to investigate the possible mechanism (s) underlying the anti-inflammatory properties of SCA and SCB compounds, their potential effects in inhibiting the activity of COX, a common target for anti-inflammatory drugs, have been evaluated. Interestingly, while SCA barely affected COX activity only at the lower concentration tested (0.1 µM), SCB concentration-dependently (0.1–10 µM) inhibited COX activity. It is worth noting that SCB, but not SCA, partially prevented TNFα-induced p65 NFκB nuclear translocation, i.e. an important transcription factor that regulates expression of inflammatory genes, including COX-2 [31]. Overall, the present findings suggest a diverse impact of SCA and SCB on COX synthesis and activity, but further studies are necessary to elucidate the cause of this difference. It should be pointed out, however, that glucocorticoids did not act as COX-inhibitor, thus suggesting that SCB possesses a different anti-inflammatory profile when compared to steroidal anti-inflammatory drugs. However, the results of the present study also indicate that both SCA and SCB are able to activate the glucocorticoid receptor, demonstrating a similar potency. The effects of the compounds were, however, lower than that of dexamethasone (0.1 µM), used as a reference glucocorticoid receptor agonist. Although the one presented is an in vitro preliminary study, these results suggest a lower efficacy of SCA and SCB in activating the glucocorticoid receptors in respect to classical corticosteroids. As the two compounds display additive anti-inflammatory mechanisms (e.g. antioxidant properties; COX-inhibition), it is possible to speculate that they maintain a relevant anti-inflammatory activity in vivo, but with a better tolerability profile than glucocorticoids. This promising scenario, however, must be confirmed in a further extensive in vivo study.

Finally, as both anti-inflammatory and antioxidant agents display neuroprotective properties, we also investigated the effects of SCA and SCB on glutamate-induced neurotoxicity. The neurotoxicity associated with high brain concentrations of the excitatory amino acid neurotransmitter glutamate has been postulated to participate in the pathogenesis of the neuronal cell loss associated with several neurological disorders, and compounds able to counteract glutamate-induced excitotoxicity have been largely investigated as potential drugs for these pathologies [32]. As expected, a 24 h exposure to glutamate induced a reduction in cell viability in primary cultures of rat cerebral cortex neurons [33]. Interestingly, the pre-treatment with SCA or SCB partially reduced the glutamate-induced neurotoxicity, thus suggesting possible neuroprotective properties of the two compounds. On the contrary, the glucocorticoid receptor agonist betamethasone did not affect glutamate-induced neurotoxicity in primary cultures of rat cerebral cortex neurons. Although still at a preliminary stage, these findings suggest a possible relevance of the two compounds in the treatment of neurodegenerative diseases.

In conclusion, the present study has shown that SCA and can be considered as non-genotoxic for Ames test; furthermore, studies on antioxidant properties have shown that both compounds presented a very high antioxidant capacity, while the control (cortisone) had not antioxidant capacity. Regarding the anti-inflammatory activity both compounds inhibited the TNFα-stimulated expression and secretion of NFkB target cytokines, also inhibited COX activity and can activate the glucocorticoid receptor. Finally, SCA and SCB display neuroprotective properties. Overall, these findings further confirm the different biological profile of SCA and SCB from that of glucocorticoids and support the development of specific preclinical studies aimed at investigating the potential beneficial effects of the two compounds in different pathological conditions, including chronic inflammation and neurodegenerative disorders.

## Materials and methods

### Bacterial strains

*Rhodococcus rhodnii* (DSM 43960) was purchased from Leibniz Institute DSMZ-German Collection of Microorganisms and Cell Cultures GmbH company. The master cell bank of *Rhodococcus rhodnii* was maintained at − 20 °C in cryovials in Plate count broth medium (1 mL) mixed with glycerol (0.5 mL) as a cryoprotectant agent. The working cell bank was conserved at 4 °C in plate count agar (PCA) slants for 6 months and used for seed cultures.

*Salmonella typhimurium* mutant TA98 and TA100 strains purchased by Molecular Toxicology Inc. (Boone, NC, USA; moltox.com).

### Synthesis of SCA and SCB through biotransformation of cortisone

The biotransformation of cortisone (Merck, Germany) with *Rh. rhodnii* DSM 43,960 was performed as previously described by Zappaterra et al. [15]*.* A loopful *of Rhodococcus rhodnii* from a culture on Plate Count Agar (PCA) (Merck, Germany) containing glucose (1 g/L), yeast extract (2.5 g/L), tryptone (5 g/L) and agar (15 g/L) was inoculated in 50 mL Erlenmeyer flasks filled with 20 mL of sterile Plate Count Broth (PCB) (Merck, Germany) containing glucose (1 g/L), yeast extract (2.5 g/L) and tryptone (5 g/L) and incubated at 30 °C and 110 rpm in an orbital shaker for 48 h. The whole cultures were introduced into 500 mL Erlenmeyer flasks containing 200 mL of sterile PCB, and after 48 h incubation at 30 °C and 110 rpm, cortisone (0.2 g) in DMSO (2 mL) was added and the culture was maintained in the same conditions. After 24 h the cells were removed by centrifugation (5242 RCF, 20 min) and the supernatant was extracted with ethyl acetate (3 × 200 mL). The organic layer was dried over anhydrous Na_2_SO_4_, the solvent evaporated and the crude mixture purified on a chromatographic column (silica gel, ethyl acetate as eluent).

### Mutagenicity assay (Ames test)

Mutagenicity assay was performed following the plate incorporation method with the histidine-requiring *Salmonella typhimurium* mutant TA98 and TA100 strains. All strains (100 µl per plate of fresh overnight cultures) were checked with and without the addition of 0.5 mL of a 5% S9 exogenous metabolic activator (S9 mix). The lyophilized post-mitochondrial supernatant S9 mix (Aroclor 1254-induced, Sprague–Dawley male rat liver in 0.154 M KCl solution, Molecular Toxicology, Inc., Boone, NC, USA), commonly used for the activation of pro-mutagens to mutagenic metabolites, was stored at− 80 °C before use. The concentration tested for all the samples were 1, 5, 10, 50, 100 µL/plate of a stock solution 100 mg/mL.

A fully-grown culture of the appropriate tester strain (0.1 mL) was added to 2 mL Molten Top Agar (0.6% agar, 0.6% NaCl, 0.5 mM L-histidine/biotin solution) at 46 °C, together with 0.1 mL of each sample solution at different concentrations, and 0.5 mL S9 mix for assays with metabolic activation or 0.5 mL of phosphate buffer pH 7.4 to test without metabolic activation. The ingredients were thoroughly mixed and poured onto minimal glucose agar plates (1.5% agar in 2% Vogel–Bonner medium E with 5% glucose solution).

DMSO was used as a negative control (100 µl/plate). Positive controls were prepared as follows: 2-aminoanthracene (2 µg/plate) and 2-nitrofluorene (2 µg/plate) for TA98 with and without metabolic activator (S9 mix) respectively; 2-aminoanthracene (2 µg/plate) and sodium azide (2 µg/plate) for TA100, with and without metabolic activator (S9 mix) respectively. The plates were incubated at 37 °C for 72 h and then the his^+^ revertants were checked and counted using a Colony Counter 560 Suntex (Antibioticos, Italy). A sample was considered mutagenic when the observed number of colonies was at least twofold over the spontaneous level of revertants [21, 22, 34]. All determinations were made in triplicate. Relative standard deviations were computed using the statistical software STATISTICA 6.0 (StatSoft Italia srl).

### Determination of antioxidant properties

#### *Photochemiluminescence (PCL-ACL) method (Photochem*^*®*^*)*

Photochemiluminescence assay, based on the methodology of Popov and Lewin [35], was performed to measure the antioxidant activity of cortisone, SCA and SCB compounds against superoxide anion radicals generated from luminol, a photosensitizer, when exposed to UV light at λ_max_ = 351 nm. The antioxidant activity of compounds was measured using ACL kits (Analytic Jena, Jena, Germany) with a Photochem apparatus (Analytic Jena, Jena, Germany). In the PCL-ACL assay, the photochemical generation of free radicals is combined with the sensitive detection using chemiluminescence. In ACL studies, the kinetic light emission curve was monitored for 3 min and expressed as mM Trolox equivalents. The areas under the curves were calculated using the PCL soft control and analysis software. The presence of Trolox (used as a standard for the calibration curve) or any antioxidants from the samples reduces the magnitude of the PCL signal, and hence, the area calculated from the integral. The observed inhibition of the signal was plotted against the concentration of Trolox added to the assay medium. The concentration of the added sample was such that the generated luminescence during the 3 min sampling interval fell within the limits of the standard curve. For ACL assay, 2.3 mL of reagent 1 (solvent and dilution reagent, methanol), 0.2 mL of reagent 2 (buffer solution), 25 μL of reagent 3 (photosensitizer: luminol 1 mmol L^−1^), and 10 μL of standard or sample solution were mixed and measured. Luminol is used as a photosensitizer and as a detecting substance for free radicals. Trolox were used for the standard calibration curve from 0.25 to 2 nM.

#### DPPH Assay

The DPPH assay was evaluated according to Aadil (2013) and Bonetti (2016) [36, 37]. Fifty microliters of samples (cortisone, SCA and SCB) were added to 1.450 μL of 0.06 mM of methanolic DPPH· radical solution. The reaction mixture was left to stand at room temperature in the dark for 15 min. The absorbance for the sample (Asample) was measured at 515 nm against methanol blank. A control was the absorbance of DPPH· solution. The percent inhibition of DPPH· radical was calculated according to the equation:

$${\text{Percent inhibition of DPPH}} \cdot {\text{ radical}} = \left[ {{1} - \left( {{\text{Asample}}/{\text{Acontrol}}} \right)} \right] \times {1}00$$

Methanolic solutions with different Trolox concentrations (0.05 − 1 mM/L) were analyzed for calibration curve. The free radical scavenging capacity was expressed as micromolar of Trolox equivalents (TEs)/g of SCA and SCB compounds using calibration curve.

### Anti-inflammatory activity

#### Cell culture and treatments

HaCaT cells were cultured with Dulbecco’s modified Eagle’s medium High Glucose (Lonza, Milan, Italy) supplemented with 10% FBS, 100 U/mL penicillin, 100 μg/mL streptomycin and 2 mM L-glutamine as previously described [38]. Cells were maintained in a humidified incubator at 37 °C with 5% CO_2_ and incubated until the monolayer was 80% confluent. Medium was changed every 2 days.

HaCaT cells were pretreated with or without 10 μM of both SCA and SCB for 24 h. Stock solutions of the two compounds were prepared in DMSO immediately before use and diluted to the final concentration in the culture medium. In some sets of experiments, betamethasone-17-valerate (10 µg/mL) served as a positive control; dimethyl sulfoxide (DMSO) served as a solvent control. After the pretreatments, cells were stimulated with 10 ng/mL TNFα for 1 h to induce the NFκB signaling pathway. Then, media were replaced with fresh culture medium. Cells and supernatants were collected at different time points for each set of experiments as described below.

#### Immunofluorescence

Briefly, HaCaT cells were seeded on coverslips at a density of 1 × 10^5^ cell/mL and incubated with DMEM high glucose supplemented with FBS 10%. Then, HaCaT cells were treated as indicated above. At the end of the proinflammatory stimulus with TNFα, cells were fixed in 4% paraformaldehyde in PBS for 30 min at 4 °C as previously described [39]. HaCaT cells were permeabilized for 5 min at RT with PBS containing 0.2% Triton X-100. After the blocking with 1% BSA in PBS at RT for 1 h, HaCaT cells were incubated with p65 NF-κB primary antibody (1:100, sc-372; Santa Cruz Biotechnology, Inc., Dallas, Texas, USA) in PBS containing 0.5% BSA at 4 °C overnight. After washing, coverslips were incubated with appropriate secondary antibody for 1 h at RT. Nuclei were stained with 1 μg/mL DAPI (Sigma- Aldrich, Italy) for 1 min. Coverslips were mounted onto glass slides using anti-fade mounting medium 1,4 diazabicyclooctane (DABCO) in glycerin. Images were acquired with Leica AF CTR6500HS (Microsystems). Negative controls were performed by omitting primary antibodies. Fluorescence was examined using an Epifluorescence microscope (Nikon Eclipse E800; Nikon Corporation, Surrey, UK) equipped with a plan apochromat 100 × 0.5 − 1.3 oil immersion objective and a mercury lamp source.

#### Real-time PCR

Quantitative real-time PCR was carried out as described in detail previously [40]. Briefly, HaCaT cells were treated as indicated above and harvested for RNA extraction 3 h after the end of the proinflammatory stimulus with TNFα. Total RNA was extracted using an AURUM total RNA Mini Kit with DNase digestion (Bio-Rad, Italy). One microgram of total RNA for each condition was used to synthesize cDNA using the iScript cDNA Synthesis Kit (Bio-Rad, Italy). Quantitative real-time PCR (qPCR) was performed using SYBR green on the CFX Multicolor real-time PCR detection system (Bio-Rad, Italy). Forward and reverse primers used are: 5′-GGTGCAGTTTTGCCAAGGAG-3′ and 5′-TTCCTTGGGGTCCAGACAGA-3′ respectively. Ribosomal proteins L13a (RPL13a) and L11a (RPL11a) and GAPDH were used as the endogenous controls. Forward and reverse primers are: RPL13a, 5′-CCTAAGATGAGCGCAAGTTGAA-3’ and 5′-CCACAGGACTAGAACACCTGCTAA-3’; RPL11a, 5′-TGCGGGAACTTCGCATCCGC-3′ and 5′-GGGTCTGCCCTGTGAGCTGC-3’; GAPDH, 5′-TGACGCTGGGGCTGGCATTG-3′ and 5′-GGCTGGTGGTCCAGGGGTCT-IL-8 3, F: 5′-GGTGCAGTTTTGCCAAGGAG-3′ and R: 5′-TTCCTTGGGGTCCAGACAGA-3′. The primer pairs were obtained from the Real-Time PCR GenBank Primer and Probe Database Primer Bank, RTPrimerDB. Real-time PCR was initiated with a 3-min hot-start denaturation step at 95 °C and then performed for 40 cycles at 95 °C for 3 s and 60 °C for 5 s. Samples were compared using the relative cycle threshold (CT). After normalization to more stable mRNA RPL13a, RPL11a, and GAPDH, the fold increase or decrease was determined with respect to control, using the formula 2 − ΔΔCT, where ΔCT is (gene of interest CT) (reference gene CT), and ΔΔCT is (ΔCT experimental) (ΔCT control).

#### IL-1α levels

IL-1α secretion, after the treatment with 10 and 20 µM of each compound and together at the same concentrations, was assayed by a commercially available ELISA kit (Thermo Scientific, Italy). At the end of the treatments, HaCaT cells were incubated for other 24 h. Then, cell-free supernatants were collected and assayed for IL-1α using a commercial ELISA kit, according to the manufacturer's instructions. The optical absorbance was measured with a spectrophotometer microplate reader at 450 nm and correction at 530 nm. Final concentrations were calculated by interpolation with a standard curve of Recombinant Human IL-1α provided by the kit and results expressed as pg/mL.

### Cyclooxygenase inhibitory assay

Cyclooxygenase (COX) activity assay was determined by using a fluorescence COX activity assay kit (Cayman, No. 700200) for detecting COX-1 and COX-2 activity in purified enzyme and tissue homogenate preparations. The assay was conducted by monitoring the appearance of fluorescent compound resofurin in accordance to the supplier recommendation. Aspirin (acetylsalicylic acid; 50 µM) served as positive control. The test compounds were dissolved in 1–2% DMSO (v/v) at 4 different concentrations (0.1, 1, 10 and 100 µM) and added to a purified COX preparation. A 1–2% DMSO solution was added to control plates. The plates were shaken for a few seconds and incubated for 5 min at 25 °C. The fluorescence was measured by using an excitation wavelength of 540 nm and an emission wavelength of 590 nm. Average fluorescent values were calculated for all the samples (n = 3/group) in order to determine the percentage of inhibition in respect to control (i.e. untreated) plates.

### Glucocorticoid receptor transactivation assays

Glucocorticoid receptor transactivation assays kit was obtained from Indigo Biosciences (Human Glucocorticoid Receptor Reporter Assay Kit, Product #IB0020-32; Indigo Biosciences, State College, PA, USA) and was utilized to assess the activation of human glucocorticoid receptor by SCA and/or SCB. Briefly, stocks of the tested compounds were prepared and diluted in a medium provided by the manufacturer. Frozen reporter cells provided in the assay kit were thawed and compound dilutions were added immediately. Cells were incubated for 24 h and the activation response was measured on a luminometer (Perkin-Elmer, MA, USA). The cells consisted of non-human mammalian cells engineered by Indigo Biosciences to provide constitutive high-level expression of full length, unmodified human glucocorticoid receptor (NR3C1). The nonhuman mammalian reporter cells included a luciferase reporter gene functionally linked to a human glucocorticoid receptor-responsive promoter. The cells are engineered so that only interactions with the human receptor will induce luciferase expression in the treated reporter cells to quantitate nuclear receptor activation. Positive control ligand performance was measured by the manufacturer and provided in the technical manuals thus allowing accurate comparison for assay performance. Additionally, the control ligands of the receptors (dexamethasone; 0.1 µM) were tested on the same test plates (to allow statistical analysis) with the tested compounds and controls.

### Effects of *SCA* and *SCB* on glutamate-induced neurotoxicity

#### Primary cultures of rat cortical neurons

Primary cultures of cortical neurons have been prepared from 1 day-old male rats [41]. After re-suspension in the plating medium, the cells were counted and then plated on poly-l-lysine (5 μg/mL)-coated dishes at a density of 2.5 × 10^6^ cells/dish or on poly-l-lysine (5 μg/mL)-coated 24-well plates at a density of 0.5 × 10^6^ cells/dish. The plating medium consisted of Eagle's Basal Medium supplemented with 10% inactivated fetal calf serum, 25 mM KCl, 2 mM glutamine and 100 μg/mL gentamycine. Cultures were grown at 37 °C in a humidified atmosphere of 5% CO_2_/95% air. Cytosine arabinoside (10 μM) was added within 24 h of plating to prevent glial cell proliferation [41]. The cultures were maintained for 8 days in vitro before experiments.

#### Glutamate-induced neurotoxicity

Following the removal of the growth medium, the cultures were exposed for 10 min to glutamate 3 μM in a Mg^2+^-free Krebs–Ringer bicarbonate buffer at 37 °C in a 5% CO_2_/95% air atmosphere. The cultures were then returned to the incubator in their growth medium. 24 h later, the glutamate-induced neurotoxicity was evaluated by measuring the neuronal viability. The different drugs tested were added 15 min before glutamate exposure and were present until the end of the experiment.

#### Evaluation of the neuronal viability by MTT reduction assay

The integrity of mitochondrial enzymes in viable neurons was determined with a colorimetric assay using 3-[4,5-dimethylthial-2-yl]-3, 5-diphenyl-tetrazolinium bromide (MTT) salt, as described by Mosdam [42]. In live cells, mitochondrial enzymes have the capacity to transform MTT salt into MTT formazan. Briefly, MTT salt (5 mg/mL in PBS 0.1 N) was added to the neuronal cultures and incubated 4 h at 37 °C. The precipitated dye was dissolved in isopropanol with 1 M HCl and colorimetrically (absorbance at 570 nm) quantified. The results were expressed as values of absorbance at 570 nm.

### Statistical analysis

For each of the variables tested, statistical analysis was performed using one-way or two-way ANOVA followed by Bonferroni’s multiple comparison test. A significant effect was indicated by a P-value < 0.05. Data are expressed as mean ± SEM of triplicate determinations obtained from three independent experiments.

## Data Availability

The data that support the findings of this study are in this published article and its supplementary information file and available from the corresponding author upon reasonable request.

## Code availability

Not applicable for that section.
